# Expression and Function of Ephrin-B1 and Its Cognate Receptor EphB2 in Human Abdominal Aortic Aneurysm

**DOI:** 10.1155/2012/127149

**Published:** 2012-07-05

**Authors:** Aiji Sakamoto, Masaaki Kawashiri, Hatsue Ishibashi-Ueda, Yuka Sugamoto, Tsuyoshi Yoshimuta, Takeo Higashikata, Hitoshi Ogino, Hayato Tada, Tetsuo Konno, Kenshi Hayashi, Masakazu Yamagishi

**Affiliations:** ^1^Department of Vascular Physiology, National Cerebral and Cardiovascular Center, Suita, Osaka 565-8565, Japan; ^2^Division of Cardiovascular Medicine, Kanazawa University Graduate School of Medicine, Kanazawa, Ishikawa 920-8640, Japan; ^3^Department of Pathology, National Cerebral and Cardiovascular Center, Suita, Osaka 565-8565, Japan; ^4^Cardiovascular Surgery, National Cerebral and Cardiovascular Center, Suita, Osaka 565-8565, Japan

## Abstract

We examined the expression of ephrin-B1 and its cognate receptor EphB2, key regulators of angiogenesis and embryogenesis, in human abdominal aortic aneurysm (AAA) and analyzed their functional roles in cell migration. From 10 patients (9 males and 1 female; age, 68.5 ± 2.4) who underwent vascular surgery for AAA, we obtained AAA and adjacent control tissues. Using real-time RT-PCR, we analyzed expression of ephrin-B1 and EphB2. We also histologically localized these molecules in AAA tissues. Finally, effects of ephrin-B1 and EphB2 on inflammatory cell chemotaxis were examined by cell migration assay. Expression levels of ephrin-B1 (0.410 ± 0.046 versus 1.198 ± 0.252, *P* = 0.027) and EphB2 (0.764 ± 0.212 versus 1.272 ± 0.137, *P* = 0.594) were higher in AAA than normal control. Both ephrin-B1 and EphB2 were expressed in macrophages, T lymphocytes, and endothelial cells within AAA. In chemotaxis assay, ephrin-B1 and EphB2 inhibited mononuclear-cell chemotaxis induced by stromal derived factor-1 down to 54.7 ± 12.7% (*P* = 0.01) and 50.7 ± 13.1% (*P* = 0.01), respectively. These data suggest that ephrin-B1 and EphB2 might be functional in human adult inflammatory cells and involved in the pathogenesis of AAA, although specific roles of these molecules should further be sought.

## 1. Introduction

Abdominal aortic aneurysm (AAA) has high risk for aortic rupture and constitutes one of the major causes of elderly death [1], sometimes being associated with coronary ectasia [2]. Compared to occlusive atherosclerosis such as carotid atheroma, AAA affects much more extensive layers of blood vessels but shares some pathogenic aspects such as inflammatory cell accumulation [3]. Genetically engineered mouse models for AAA have elucidated key molecules for the pathogenesis of AAA [4]. For example, some matrix metalloproteinases (MMPs) are upregulated and expressed in macrophages within AAA, which is likely to cause medial degeneration in AAA [5] with or without physiological stress such as hypoxia [6]. However, our understanding on the molecular and cellular pathogenesis of AAA is still limited, especially in cases of humans.

Recently, we have found that ephrin-B1 and its cognate receptor EphB2, the key regulators of angiogenesis and embryogenesis, are upregulated and predominantly expressed in macrophages and T-lymphocytes in human carotid atherosclerotic plaque [6]. Ephrin-B1 and EphB2 belong to ephrin and Eph family of genes consisting 21 members, which are expressed ubiquitously in embryonic tissues and involved in morphogenesis by regulating cell adhesion and migration [7, 8]. Therefore, we hypothesized that ephrin-B1 and EphB2 might be also involved in the pathogenesis of AAA and set out to analyze the expression of these molecules in human AAA and their modulatory effects on chemotaxis of inflammatory cells.

## 2. Methods

### 2.1. Patients

The experimental protocol complied with the principles of the Declaration of Helsinki and was approved by the ethical committee of National Cerebral and Cardiovascular Center. Written informed consent was obtained from all the 10 patients who underwent elective graft replacement surgery for AAA (9 males and 1 female; age, 68.5 ± 2.4) [9]. None of the AAA patients suffered from clinically unstable state such as aortic rupture before surgery. The diameter of AAA measured by computed tomography ranged from 38 to 80 mm (55.3 ± 3.5 mm). The prevalence of risk factors for AAA was as follows: hypertension in 9, hyperlipidemia in 8, smoking in 7, and diabetes mellitus in 2 out of 10 patients.

### 2.2. Aortic Tissue Sampling

During graft replacement for AAA, a strip of aortic wall that contained the dilated region and lacked mural thrombus was carefully excised. An infra-renal aortic strip which contained minimal atherosclerotic changes without dilation was also obtained from five patients as control. All the samples were quickly frozen in liquid nitrogen and stored at −80°C until extraction of RNA. A part of the tissue was fixed with non-formalin-fixative solution, Histochoice (Amresco, Solon, OH), and embedded in paraffin.

### 2.3. Antibodies and Reagents

For immunohistochemistry, we used the following mouse monoclonal antibodies: anti-CD68 (clone KP1) (Dako, Glostrup, Denmark) and anti-CD8 (clone 4B11) (Novocastra, Newcastle, UK). The following polyclonal antibodies were employed: rabbit anti-ephrin-B1 (Santa Cruz, Santa Cruz, CA); goat anti-EphB2 (Sigma, St. Louis, MO); rabbit anti-von Willebrand Factor (vWF) (Dako); rabbit anti-CXCR-4 (Sigma-Aldrich, St. Louis, MO). Biotinylated swine anti-goat IgG (Dako) was also used. The following recombinant proteins were used: human stromal-derived factor-1 (SDF-1) (PeproTech, London, UK); human monocyte chemotactic protein-1 (MCP-1) (BioLegend, San Diego, CA); mouse ephrin-B1-IgG-Fc chimera and mouse EphB2-IgG-Fc chimera (R&D Systems, Minneapolis, MN); IgG-Fc (Athens Research & Technology, Athens, GA).

### 2.4. Quantitative Reverse Transcription-Polymerase Chain Reaction (Quantitative RT-PCR)

Total RNA was isolated from the aortic specimens using Isogen reagent (Nippon Gene, Tokyo, Japan) according to the manufacturer's instructions [10]. Concentration of the RNA was measured with spectrophotometer and its integrity was confirmed visually on 1.2% denaturing agarose gel electrophoresis stained with SYBR Green dye (Invitrogen, Carsbad, CA). Then, 10 *μ*g of total RNA was treated with DNA-free agent (Ambion, Austin, TX) and converted to cDNA using random primers and SuperScript II reverse transcriptase (Invitrogen). Quantitative RT-PCR was performed using TaqMan reagents on ABI Prism 7700 Sequence Detection System (PE Applied Biosystems, Foster City, CA). Assay-on-Demand cocktails (PE Applied Biosystems) were used for ephrin-B1 (Hs00270004_m1) and EphB2 (Hs00362096_m1). For GAPDH, predeveloped TaqMan assay reagent was used. The expression levels of ephrin-B1 or EphB2 were normalized to those of GAPDH as described previously [5, 11].

### 2.5. Immunohistochemistry

The paraffin-embedded specimens of AAA were sectioned in 3 *μ*mthickness. These sections were deparaffinized with Neo-Clear (Merk, Darmsdat, Germany). For histopathological evaluation, the sections were stained with hematoxylin and eosin (H&E). For double immunohistochemical staining of ephrin-B1 and CD68, ephrin-B1 and CD8, ephrin-B1 and vWF, or ephrin-B1 and CXCR-4, the sections were first incubated with anti-ephrin-B1 antibody (1 : 300 dilution) and indirectly stained as brown precipitates using peroxidase-coupled Envision system (PO-Envision, Dako) and 3, 3′-diaminobenzidine (DAB) as substrate. Subsequently, the same sections were incubated with anti-CD68 (1 : 300 dilution), anti-CD8 (1 : 40 dilution), anti-vWF (1 : 200 dilution), or anti-CXCR4 (1 : 100 dilution) antibodies and stained as red precipitates using alkaline phosphatase-coupled Envision system (AP-Envision, Dako) and New Fuchsin (Dako) as substrate.

For double immunostaining of EphB2 and CD68, EphB2 and CD8, EphB2 and vWF, or EphB2 and CXCR-4, sections were first incubated with goat anti-EphB2 antibody (1 : 50 dilution) and processed with peroxidase-coupled Histofine MAX-(G) system (Nichirei, Tokyo, Japan) and DAB. The same sections were then incubated with anti-CD68, anti-CD8, anti-vWF, or anti-CXCR4 antibodies and processed with AP-Envision and New Fuchsin. Before the double immunohistochemistry for ephrin-B1 and CD8 or EphB2 and CD8, the sections had been autoclaved in 0.01 mol/L citrate buffer (pH 6.0) for 5 minutes at 121°C to unmask CD8 antigen. For double immunostaining of CD68 and CXCR-4, the sections were first incubated with anti-CD68 antibody and processed with PO-Envision and DAB. The same sections were then incubated with anti-CXCR-4 antibody and processed with AP-Envision and New Fuchsin. All these preparations were counterstained with hematoxylin, mounted with Neo-Mount (Merk), and examined with Axiophot2 light microscope equipped with AxioCam CCD camera (Carl Zeiss, Hallbergmoos, Germany).

### 2.6. RT-PCR for Ephrin-B1 and EphB2 in Peripheral Blood Mononuclear Cells

Human peripheral blood mononuclear cells (PBMC) were prepared from forearm venous blood of healthy adult volunteers by density-gradient centrifugation through Lymphoprep (Axis-Shield PoC AS, Oslo, Norway). From the human PBMC, total RNA was isolated with Isogen reagent, which was cleared of contaminating genomic DNA and reversely transcribed to cDNA using QuantiTect Reverse Transcription Kit (Qiagen, Hilden, Germany). For RT-PCR, the forward and reverse primers were designed within a single exon  of  ephrin-B1  (exon  5)  or  EphB2  (exon  6) : ephrin-B1, 5′-GTCCTACTACTGAAGCTACG-3′/5′-CTCTTGGACGATGTAGACAG-3′;  EphB2, 5′-GCAGTGTCCATCATGCATC-3′/5′-AGTACTGCAGCTCATAGTCC-3′.

As negative and positive controls, the DNA-cleared RNA without reverse transcription and human genomic DNA (50 nM; Clontech, Palo Alto, CA) were used, respectively. The reaction mixture was assembled to a total volume of 10 *μ*L as follows: 6.65 *μ*L water, 1.0 *μ*L 10 × Ex Taq buffer, 0.8 *μ*L dNTP mixture (comprising 2.5 mM of each nucleotide), 1.0 *μ*L forward and reverse primer mixture (5 *μ*M of each primer), 0.5 *μ*L template, and 0.05 *μ*L Ex Taq polymerase (TaKaRa, Tokyo, Japan). The PCR was carried out with preheating (94°C for 2 min) and 35 cycles of amplification (94°C for 20 s, 60°C for 30 s and 72°C for 40 s). The PCR products were subjected to acrylamide gel electrophoresis and stained with Ethidium bromide. The experiments were performed at least three times and the representative data are shown.

### 2.7. Cell Migration Assay

Boyden chamber cell migration assay was performed using 24-well Transwell plates which contain, in each well, a chamber with 5 *μ*m porous polycarbonate membrane separating the well into upper and lower parts (Corning, Corning, NY). The chamber membranes were precoated with ephrin-B1-Fc, EphB2-Fc, or IgG-Fc as control (5 *μ*g/mL, overnight). Then the chambers were inserted into Transwell plates containing 600 *μ*L of assay buffer (RPMI1640 medium with 0.1% heat-inactivated BSA) with or without chemokines (SDF-1 or MCP-1 at 0.1 *μ*g/mL). Human PBMC (1 × 10^5^ cells in 100 *μ*L of the assay buffer) were loaded into the chamber and allowed to migrate down to the lower part of the well for 2 hours in a humidified incubator (37°C; 5% CO_2_). After the migration period, the PBMC that had passed down through the membrane were collected and counted using Z2 Coulter counter (Beckman Coulter, Fullerton, CA). Under the same condition, we also tried to use soluble recombinant ephrin-B1 and EphB2 diluted into the medium to assess the cell migration, because there were some reports that demonstrated differences in results of cell migration [12].

### 2.8. Statistical Analysis

The results of Quantitative RT-PCR and Boyden chamber assay were expressed as mean ± SEM of two independent experiments done in triplicate. All statistical analyses were performed by using StatView Ver.5.0 software (SAS Institute Inc, Cary, NC). A value of *P* < 0.05 was considered to be statistically significant.

## 3. Results

### 3.1. Expression of Ephrin-B1 and EphB2 in Human AAA

**Figure 1:**
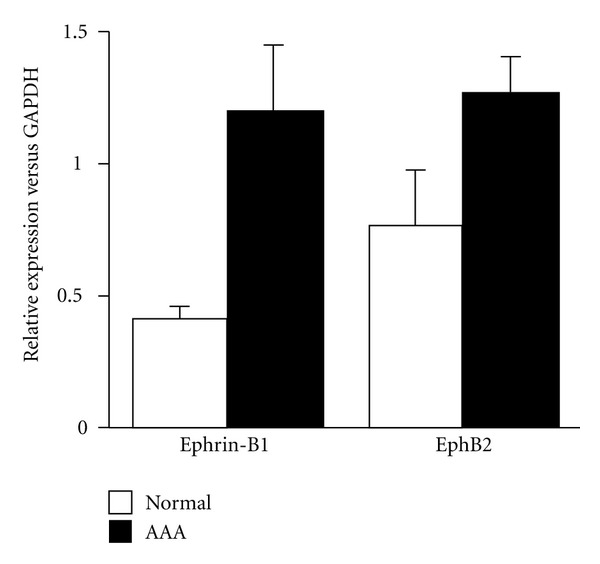
Quantitative real-time RT-PCR analysis of ephrin-B1 and EphB2 in human AAA. The results are mean ± SEM (*n* = 10) and were analysed by an unpaired Student's *t*-test.

All the total RNA isolated from AAA or control aortas maintained robust integrity on agarose gel electrophoresis, ensuring reliable analysis of gene expression. Quantitative real-time RT-PCR showed that ephrin-B1 was significantly upregulated in AAA than in control (Figure 1). As for EphB2, a trend toward higher expression in AAA was observed, although the data did not reach statistical significance.

### 3.2. Immunohistochemical Analysis of Ephrin-B1 and EphB2 in Human AAA Lesions

**Figure 2:**
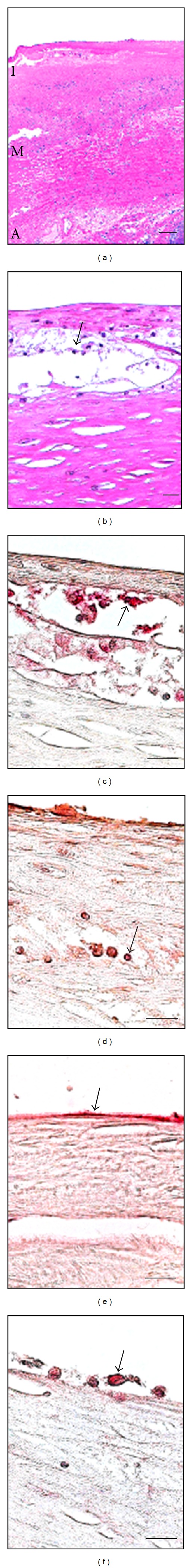
Histology of human AAA and double immunostaining of ephrin-B1 and others therein. (a) I, intima; M, tunica media; A, adventitia. Note severely destructed tunica media. Bars, 200 *μ*m. (b) Arrow indicates fatty streak in intima. Bars, 20 *μ*m.(c) and (d)Double immunostaining. Ephrin-B1 was stained with DAB (brown), the others with New Fuchsin (red). Bars, 20 *μ*m.

Each of the tissue specimens used in this study showed typical structures of AAA. All the layers of aorta were affected and the tunica media were severely damaged (Figure 2(a)). Prominent fatty streaks and inflammatory cell infiltration were observed in the intima (Figure 2(b)). Under these conditions, the presence of these inflammatory cells was apparently greater in AAA tissues than those in controls. We performed double immunohistochemical staining to clarify the precise cell types expressing ephrin-B1 or EphB2 therein, because we found that ephrin-B1 and EphB2 were expressed in various types of cells within human AAA with single immunostaining.

**Figure 3:**
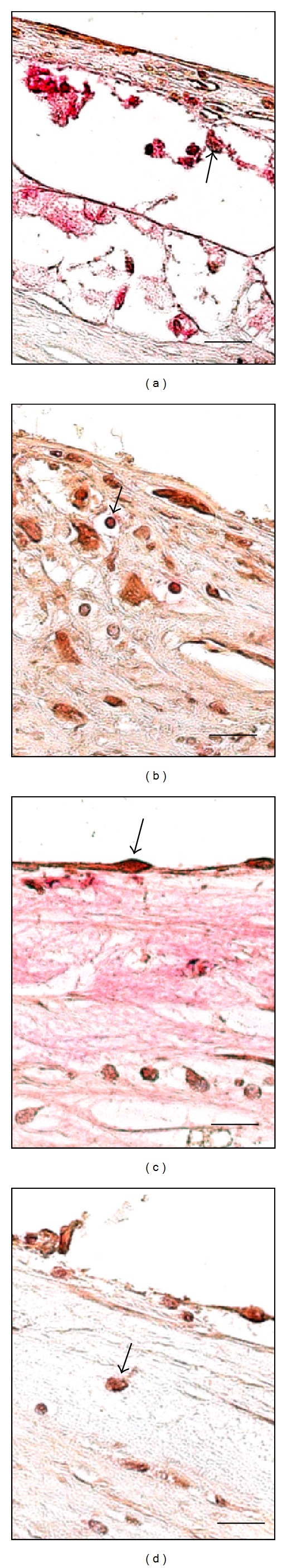
Double immunostaining of EphB2 and others in human AAA. EphB2 was stained with DAB (brown), the others with New Fuchsin (red). Bars, 20 *μ*m.

 Immunoreactivity of ephrin-B1 was found in the following cell types: macrophages positive for CD68 [13] (Figure 2(c)); lymphocytes positive for CD8 [14] (Figure 2(d)); endothelial cells positive for vWF [15] (Figure 2(e)). Similarly, EphB2 was detected in macrophages positive for CD68 (Figure 3(a)), lymphocytes positive for CD8 (Figure 3(b)), and endothelial cells positive for vWF (Figure 3(c)).

To investigate functional significance of ephrin-B1 and EphB2 on chemotaxis of macrophages or lymphocytes, we examined coexpression of these molecules with CXCR4, a receptor for SDF-1 [16] by double immunohistochemistry. Many macrophage- or lymphocyte-like cells were found to express both ephrin-B1 and CXCR4 (Figure 2(f)) or both EphB2 and CXCR4 (Figure 3(d)).

### 3.3. Functional Expression of Ephrin-B1 and EphB2 in Peripheral Blood Mononuclear Cells

**Figure 4:**
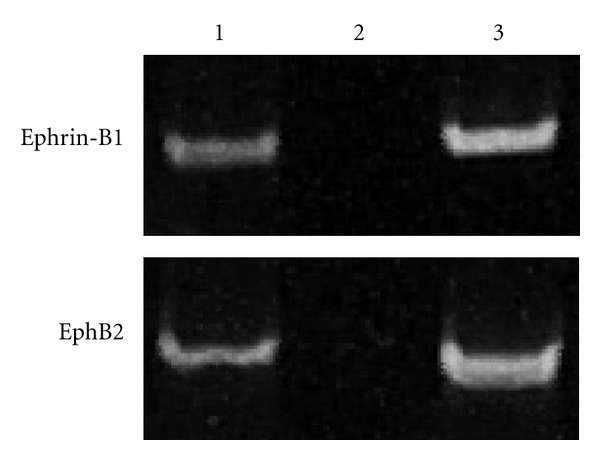
RT-PCR analysis for ephrin-B1 and EphB2 in human PBMC. Three kinds of templates were used to validate the results: lane 1, cDNA; lane 2, DNA-cleared RNA; lane 3; genomic DNA.

**Figure 5:**
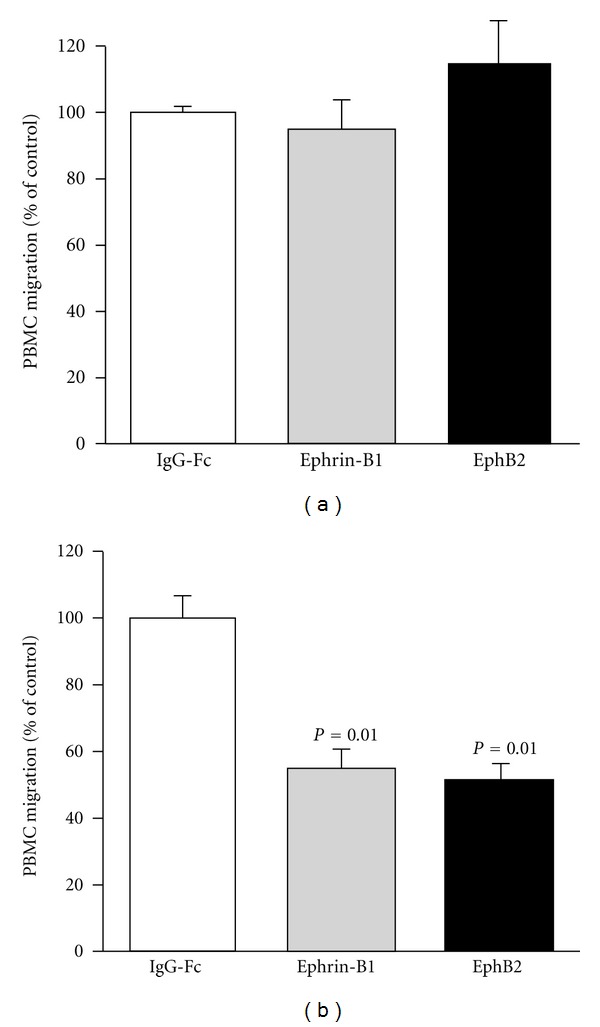
Modulatory effects of ephrin-B1 and EphB2 on transmigration of human PBMC by SDF-1. Boyden chamber assay was performed. (a) Effect of ephrin-B1 or EphB2 on spontaneous transmigration. The number of cells migrating into the well without SDF-1 was considered spontaneous transmigration. (b) Effect of ephrin-B1 or EphB2 on SDF-1-induced transmigration. With SDF-1, the increased number of cells migrating into the well was due to SDF-1-dependent transmigration. The level of PBMC migration with IgG-Fc used as control, which was 3.6% (a) and 18.5% (b) of inputs (1 × 10^5^ cells), was set at 100% for analysing the effect of ephrin-B1 or EphB2. Ephrin-B1, ephrin-B1-IgG-Fc; EphB2, EphB2-IgG-Fc. The results are mean ± SEM (*n* = 6) and were analysed by one factor ANOVA and Dunnett's test compared with IgG-Fc (control).

To confirm the expression at mRNA levels of ephrin-B1 and EphB2 on human adult inflammatory cells, we performed RT-PCR. In human PBMC, both ephrin-B1and EphB2 were robustly expressed (Figure 4). We then tested whether ephrin-B1 and EphB2 have functional importance in human PBMC, by examining the effects of ephrin-B1 and EphB2 on their chemotaxis. In Boyden chamber assay, membrane-coated ephrin-B1-Fc or EphB2-Fc chimeric proteins did not affect spontaneous migration of PBMC (Figure 5(a)). However, the chemotaxis of PBMC induced by SDF-1 was significantly inhibited by ephrin-B1-IgG-Fc and EphB2-IgG-Fc down to 54.7 ± 12.7% (*P* = 0.01) and 50.7 ± 13.1% (*P* = 0.01), respectively (Figure 5(b)). Also, MCP-1-induced chemotaxis of PBMC was significantly inhibited by ephrin-B1-IgG-Fc and EphB2-IgG-Fc down to 53.8 ± 10.9% (*P* = 0.01) and 51.3 ± 11.6% (*P* = 0.01), respectively, although no significant increases or decreases of cell migration were observed in cases of soluble ephrin-B1 and EphB2.

## 4. Discussion

We found that both ephrin-B1 and EphB2 exhibited higher expression in human AAA and were expressed in macrophages, T lymphocytes, and endothelial cells therein. Furthermore, we found that membrane-bound ephrin-B1 and EphB2 inhibited chemotaxis of human PBMC in Boyden chamber assay. Ephrins and their cognate receptor Ephs are well-known regulators in embryogenesis [7, 8] but their expressions and pathogenic roles in AAA remain unclear. Therefore, the findings of this study would give us a new clue to investigate the molecular pathogenesis of human AAA.

One major cause of higher expression of ephrin-B1 and EphB2 in AAA is probably the infiltration therein of macrophages and T-lymphocytes expressing these two genes. However, gene expression levels of ephrin-B1 and EphB2 in monocytes/macrophages, T-lymphocytes, and endothelial cells in AAA might be upregulated, which remains to be elucidated. Taken together with the fact that ephrin-A5 is upregulated in human AAA [17], wide spectrum of ephrins and Ephs [7] might be upregulated in human AAA. We further tested whether ephrin-B1 and EphB2 are functional or nonfunctional on human adult inflammatory cells. For this purpose, we examined the effects of ephrin-B1 and EphB2 on chemotaxis, because these molecules are well known to regulate migration of embryonic cells. In Boyden chamber assay, the membrane-fixed ephrin-B1 inhibited chemotaxis of human PBMC induced by SDF-1 or MCP-1, which is in line with the previous reports that ephrin-B1 inhibits SDF-1-induced chemotaxis of Jurkat T-lymphocytic [18] and THP-1 monocytic cell lines [6].

The chemotaxis of human PBMC induced by SDF-1 or MCP-1 was also inhibited by the membrane-fixed EphB2, which usually functions as receptor. This ligand-like action by EphB2 is considered so-called reverse signalling [8], like EphB2 inhibiting SDF-1-dependent migration of cerebellar granule cell of mouse embryo [19]. In addition, there are several reports which show ephrin signalling interacts with inflammatory molecules such as Rho family GTPases [20] and VCAM-1 [21]. Thus, our findings from Boyden chamber assay suggest that ephrin-B1 and EphB2 on human PBMC are functional.

Importantly, these inhibitory effects of ephrin-B1 and EphB2 were not clearly observed when we used soluble agents. This may be partially explained by a dependence of Eph receptor activation on lateral movement across the cell membrane for complete activity [12]. Pfaff et al. [22] demonstrated lateral movement of endothelial ephrinB2 from the apical surface to cell-cell junctions preceding degradation. Additionally, one might speculate that ephrins/eph receptors fixed to transwell membranes may not induce proper spatial distribution of counter-receptors/ligands for chemotaxis. However, the present data demonstrate at least the inhibitory effects of cell migration induced by SDF-1 *in vitro*.

Including cytokines and chemokines, most of the known factors that regulate the activities of macrophages or T-lymphocytes are soluble molecules which are widely spread [23]. In contrast, ephrins and Ephs are membrane-bound molecules which exert their functions locally through cell-to-cell interaction with their neighbouring cells [24]. During the pathological courses of many inflammatory diseases including AAA [25–29], various types of cell-to-cell interaction occur: macrophages-to-macrophages, macrophages-to-T-lymphocytes, macrophages-to-endothelial cells, and so on. Ephrin-B1 and EphB2, or more generally ephrins and Ephs, might locally and precisely modulate the activities of macrophages and T-lymphocytes conditioned by diffusing factors such as cytokines and chemokines. Understanding the effect of ephrinB1 or EphB2 activation on the inflammatory condition, perhaps to MMP expression or function, may make clear the role of ephrinB1 or EphB2 in this context [30]. Interestingly, we recently found that ephrin and Eph family are widely expressed in atherosclerosis-related cells in human [31]. Further study will elucidate the* in vivo* roles of ephrins and Ephs in the development of atherosclerosis, both dilated and occlusive, and other inflammatory diseases.

## 5. Conclusions

This study suggests that ephrin-B1 and EphB2, the key embryogenic regulators, might be involved in the molecular and cellular pathogenesis of human AAA. Through cell-to-cell interactions, ephrin-B1 and EphB2 might locally modulate the inflammatory cell activities conditioned by soluble mediators such as cytokines. Ephrin/Eph system, the largest receptor tyrosine kinase system in the human genome, may give us a clue to unravel yet unknown pathogenesis of AAA and to devise novel therapeutic strategies for AAA and other inflammatory diseases, although specific roles of these molecules in the development of AAA should further be sought.
